# Sudden Cardiac Arrest at the Triage of Emergency Medicine Department in a Patient With COVID-19

**DOI:** 10.7759/cureus.11795

**Published:** 2020-11-30

**Authors:** Fawaz Altuwaijri, Omar Alotaibi

**Affiliations:** 1 Emergency, King Saud University, Riyadh, SAU; 2 Medicine, King Saud University, Riyadh, SAU

**Keywords:** covid-19, post cardiac arrest care, ventricular fibrillation, severe acute respiratory syndrome coronavirus 2

## Abstract

A pneumonia outbreak with an unknown microbial etiology was reported in Wuhan, Hubei province of China, on December 31, 2019. This was later attributed to a novel coronavirus, currently called as severe acute respiratory system coronavirus 2 (SARS-CoV-2). Coronavirus disease 2019 (COVID-19) mainly affects the respiratory system and can also cause acute or chronic damage to the cardiovascular system.

We present a case of a 64-year-old female with past medical history of diabetes mellitus and hypertension who presented to the Emergency Medicine Department with shortness of breath and worsening chest discomfort, then had a ventricular fibrillation (VF) arrest while in triage, in the context of COVID-19 diagnosis.

Cardiovascular complications during the COVID-19 pandemic should be brought to medical attention; it is crucial that physicians be aware of the complications and treat it as an emergency.

## Introduction

In late December 2019, the first case of severe acute respiratory syndrome coronavirus 2 (SARS-CoV-2), also known as coronavirus disease 2019 (COVID-19) was reported in Wuhan, China [1]. Since then, the virus has spread rapidly around the world leading to a global public health crisis and declared as a pandemic by the World Health Organization in March 2020 [2]. As of this writing, globally there are more than 13 million confirmed cases and > 500,000 deaths, and in Saudi Arabia, more than 230,000 confirmed cases and > 2000 deaths [3]. The clinical features of COVID-19 are varied, ranging from asymptomatic to acute respiratory distress syndrome and multi-organ dysfunction [4], and primarily affecting the respiratory system, causing severe pneumonia [5]. One of the most frequent presentations is dyspnea and chest pain, and that can be challenging whether it is a respiratory or cardiac cause [6]. COVID-19 has been associated with a wide variety of complications during the infection. Pulmonary, thrombotic, and cardiovascular complications have been widely reported [7-8].

## Case presentation

A 64-year-old female patient with a history of diabetes mellitus and hypertension presented to our Emergency Medicine Department complaining of shortness of breath and worsening chest discomfort. Two weeks prior to presentation, she had a history of sore throat, fever, and diarrhea for three days. Later, she underwent polymerase chain reaction (PCR) testing and was diagnosed with COVID-19.

**Figure 1:**
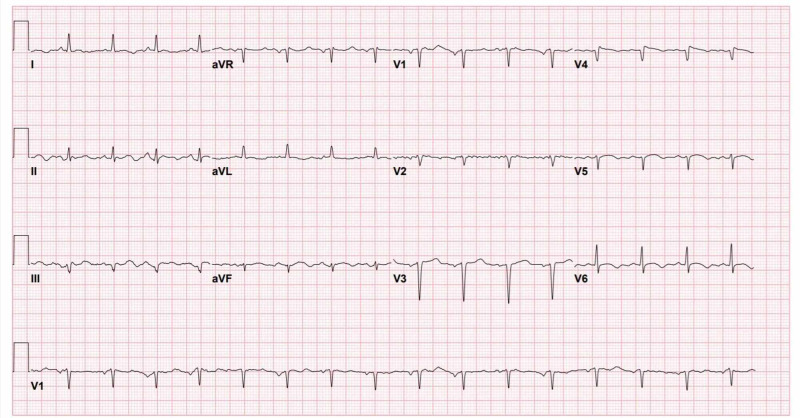
Initial ECG before cardiac arrest. ECG, electrocardiogram

**Figure 2:**
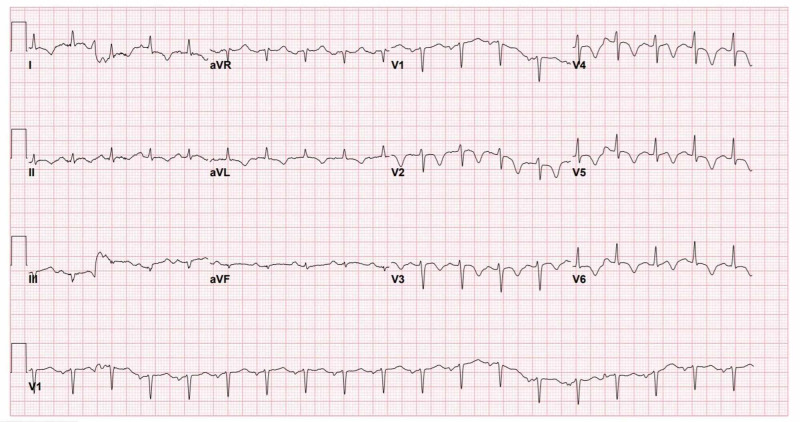
ECG done after ROSC. ECG, electrocardiogram; ROSC, return of spontaneous circulation

**Figure 3:**
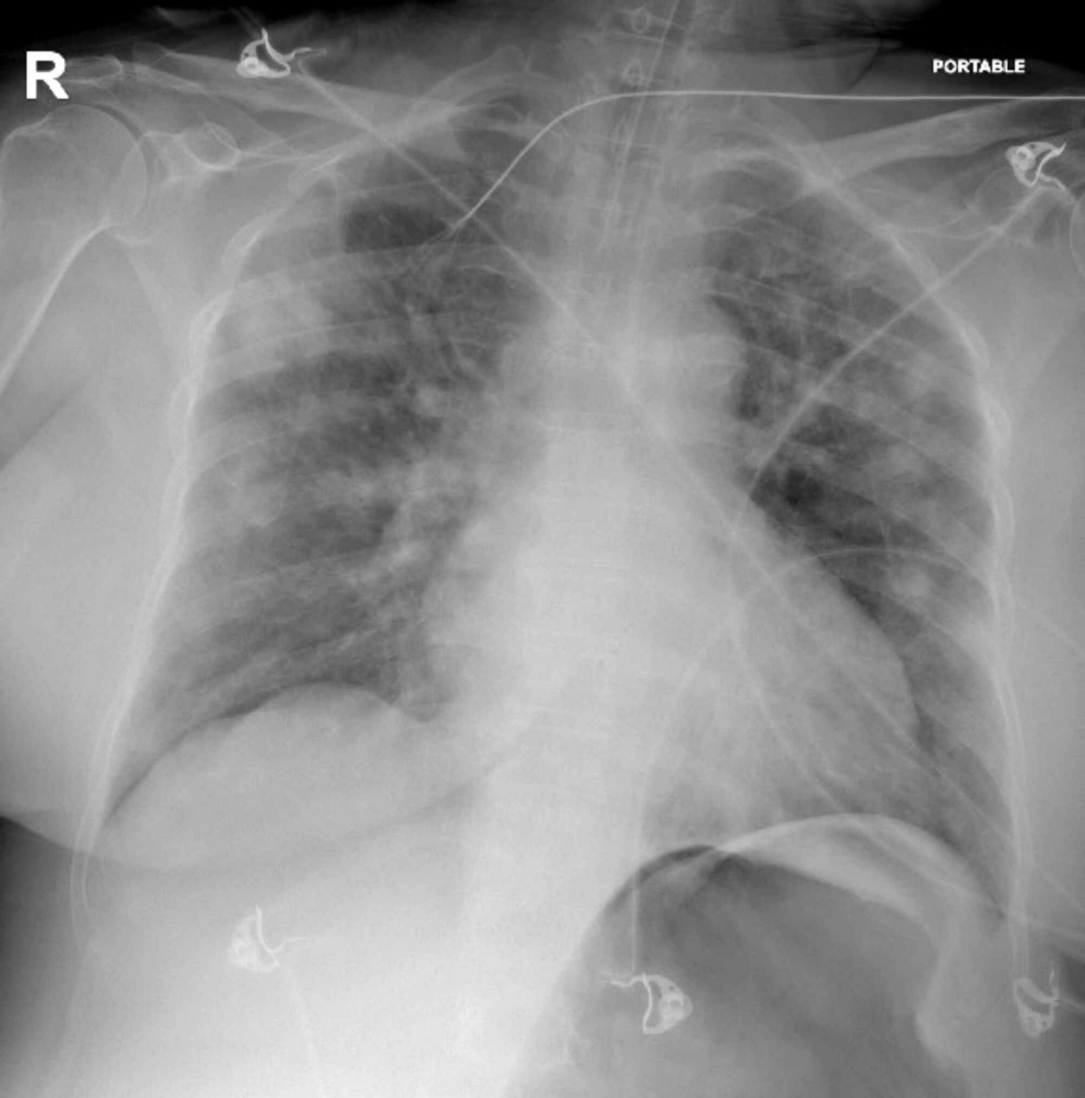
Chest X-ray shows patchy infiltrate seen bilaterally.

The patient became unresponsive just after the initial electrocardiogram (ECG) was done in triage, and advanced cardiac life support protocol was started. Initial rhythm showed ventricular fibrillation (VF). She received three shocks of 200 J for each, amiodarone 300 mg, and two doses of epinephrine 1 mg during resuscitation. Return of spontaneous circulation (ROSC) was achieved after four cycles of cardiopulmonary resuscitation around 12 min of duration, then she was intubated, sedated, and ventilated. She was vitally stable on presentation; however, post-ROSC, her initial vital signs were as follows: blood pressure of 70/41 mmHg, respiratory rate 24 breaths per minute, and oxygen saturation of 99% on the ventilator. Norepinephrine 15 mcg/min was commenced through a peripheral vein, followed by an insertion of a femoral central venous access. Unfortunately, this was complicated by vasopressor extravasation and subsequent digits ischemia, for which the plastics team was involved in her care in ICU. Pre-arrest ECG was normal (Figure 1). However, ECG done after ROSC showed evolving ST-elevation in inferior leads without reciprocal changes (Figure 2). Myopericarditis was the final diagnosis made by the cardiologists. Chest X-ray (Figure 3) showed patchy air space opacities seen overlying the bilateral lung fields which are consistent with classic COVID-19 pneumonia. The patient was transferred to the ICU, where she became hemodynamically stable on inotropes with Glasgow Coma Scale (GCS) of 7-8 due to anoxic brain injury. Admission labs were remarkable for elevated troponin-I level at 25,125 ng/L which was 20 ng/L at the time of presentation, then started to trend down, brain natriuretic peptide (BNP) of 190 pg/mL, white blood count of 15.480 x 109/L, prothrombin time (PT) of 16.3 s, activated partial thromboplastin time (aPTT) of 43.40 s, Na of 129 mmol/L, K of 5.3 mmol/L, and glucose of 16 mmol/L. Urine culture showed Pseudomonas aeruginosa, for which, IV ciprofloxacin was commenced. On hospital day 5, the patient was extubated and subsequently she is saturating well at 95%-100% at room air. On hospital day 10, she developed spiked fever (38.5°C), blood cultures were sent and empiric IV vancomycin and meropenem were started; then culture came positive for methicillin sensitive staphylococcus aureus (MSSA) and the management changed to IV cefazolin.

The patient is still admitted at the time of writing this report, transferred to medical ward with low level of consciousness and GCS of 9. She is hemodynamically stable and afebrile, and echocardiogram is planned after clearance from COVID-19, complete three-days of azithromycin and ceftriaxone, and 10 days of dexamethasone as per COVID-19 management protocol, and she is currently on aspirin as maintenance therapy for pericardial involvement, enoxaparin as deep vein thrombosis (DVT) prophylaxis, lantus, and aspart for diabetes mellitus management.

## Discussion

The SARS-CoV-2 is an enveloped, single-stranded, and positive-sense RNA virus belonging to the beta-coronaviridae family [9], and the pathophysiology of COVID-19 is not yet fully understood; nevertheless, it has been confirmed that SARS-CoV-2 enters the host cell via fusion to angiotensin-converting enzyme 2 (ACE2) receptor, which is expressed in multiple organs [10]. The clinical presentation of most of the COVID-19 patients incudes fever, difficulty breathing, and fatigue [6], however, the minority of patients present with chest pain and palpitations as an evidence of myocardial injury [11-12]. Initial evaluation tests include complete blood count, liver function test, renal function test, inflammatory markers, coagulation profile and cardiac enzymes, an elevation of troponin level not only related to myocardial infarction in COVID-19 patients.

The mechanisms behind COVID-19 impact on cardiovascular system are not yet confirmed; however, there are several possibilities for direct or indirect effect to the heart, it can cause myocardial infarction by ACE2-related signaling pathway, oxygen demand-supply mismatch due to respiratory failure or hypoxia, systemic inflammatory response syndrome with cytokine storm create immune-mediated process which lead to myocarditis [10], or it can affect the conduction system that leads to cardiac arrhythmia. A recent study demonstrated various types of cardiac arrhythmias associated with COVID-19 after hospitalization, which includes atrioventricular block, atrial fibrillation, polymorphic ventricular tachycardia, and pulseless electrical activity. However, the onset of development of new arrhythmia varies after COVID-19 infection [12], and new onset of ventricular fibrillation (VF) has been reported in COVID-19 patients [13]. The most frequent cardiovascular complications in hospitalized patients with COVID-19 are heart failure, myocardial injury, arrhythmias, and acute coronary syndrome [14]. 

In this case, we illustrate one of the manifestations of COVID-19 infection in the cardiovascular system; the patient rapidly developed VF arrest upon presentation. The clinical presentation, laboratory results, and post-arrest ECG are consistent with myopericarditis [15]. Echocardiogram and cardiovascular magnetic resonance (CMR) are mandatory to assess myocardial involvement. These tests were not done for our patient as a precaution to minimize exposure of healthcare workers, and that is considered as a limitation in our report.

## Conclusions

During COVID-19 pandemic, attending physicians should rule out cardiovascular complications in acute settings and in case of an arrest standard advanced cardiac life support with appropriate personal equipment should be performed for all patients in whom COVID-19 is suspected. Baseline ECG should be performed at the time of presentation. Myopericarditis is suspected in patients with localized ST-elevation without reciprocal changes with elevation of cardiac enzymes.

## References

[1] Singhal T (2020). A review of coronavirus disease-2019 (COVID-19). Indian J Pediatr.

[2] Mahase E (2020). Covid- 19: WHO declares pandemic because of “alarming levels” of spread, severity, and inaction. BMJ.

[3] (2020). World Health Organization. Coronavirus disease (COVID-19) Situation Report - 177 [Internet]. https://www.who.int/docs/default-source/coronaviruse/situation-reports/20200715-covid-19-sitrep-177.pdf.

[4] Abduljalil JM, Abduljalil BM (2020). Epidemiology, genome, and clinical features of the pandemic SARS-CoV- 2: a recent view. N Microb N Infect.

[5] Rothan HA, Byrareddy SN (2020). The epidemiology and pathogenesis of coronavirus disease (COVID-19) outbreak. J Autoimmun.

[6] Grant MC, Geoghegan L, Arbyn M (2020). The prevalence of symptoms in 24,410 adults infected by the novel coronavirus (SARS-CoV-2; COVID- 19): a systematic review and meta-analysis of 148 studies from 9 countries. PLoS One.

[7] Ranucci M, Ballotta A, Di Dedda U (2020). The procoagulant pattern of patients with COVID-19 acute respiratory distress syndrome. J Thromb Haemost.

[8] Madjid M, Safavi-Naeini P, Solomon SD, Vardeny O (2020). Potential effects of coronaviruses on the cardiovascular system: a review. JAMA Cardiol.

[9] Chan JFW, Kok KH, Zhu Z (2020). Genomic characterization of the 2019 novel human-pathogenic coronavirus isolated from a patient with atypical pneumonia after visiting Wuhan. Emerg Microbes Infect.

[10] Imazio M, Klingel K, Kindermann I (2020). COVID-19 pandemic and troponin: indirect myocardial injury, myocardial inflammation or myocarditis?. Heart.

[11] Liu K, Fang YY, Deng Y (2020). Clinical characteristics of novel coronavirus cases in tertiary hospitals in Hubei Province. Chin Med J.

[12] Kochav SM, Coromilas E, Nalbandian A (2020). Cardiac arrhythmias in COVID-19 infection. Circ Arrhythm Electrophysiol.

[13] Elsaid O, McCullough PA, Tecson KM (2020). Ventricular fibrillation storm in coronavirus 2019. Am J Cardiol.

[14] Kunutsor SK, Laukkanen JA (2020). Cardiovascular complications in COVID-19: a systematic review and meta-analysis. J Infect.

[15] Rivera-Morales MD, Pell R, Rubero J (2020). Acute myopericarditis in the post COVID-19 recovery phase. Cureus.

